# Exploratory Changes in Surfactant Protein D During Intermittent Hypoxia and Modulation by Galectin-3 Inhibition

**DOI:** 10.3390/arm94030027

**Published:** 2026-04-24

**Authors:** Saad Al-Anazi, Yasser A. Alshawakir, Syed Shahid Habib, Hayam Gad, Asma F. Alotaibi, Alanoud T. Aljasham, Wajd Ahmed Althakfi, Mohamed A. Mekhtiche, Abeer Abdulmoati Al-Masri

**Affiliations:** 1 Department of Physiology, College of Medicine, King Saud University, Riyadh 11461, Saudi Arabia; sshahid@ksu.edu.sa (S.S.H.); hgad@ksu.edu.sa (H.G.); aelmasri@ksu.edu.sa (A.A.A.-M.); 2 Azeer Medical Company, Jeddah 21382, Saudi Arabia; 3 Experimental Surgery and Animal Laboratory, Prince Naif for Health Research Center (PNHRC), College of Medicine, King Saud University, Riyadh 11451, Saudi Arabia; yalshawakir@ksu.edu.sa; 4 Department of Pharmacology and Toxicology, College of Pharmacy, King Saud University, Riyadh 11451, Saudi Arabia; asma-otibi@hotmail.com; 5 Department of Clinical Laboratory Sciences, College of Applied Medical Sciences, King Saud University, Riyadh 12372, Saudi Arabia; aaljasham@ksu.edu.sa; 6 Histopathology Unit, Department of Pathology, College of Medicine, King Saud University Medical City, Riyadh 11472, Saudi Arabia; wajdalthakfi@gmail.com; 7 Department of Computer Engineering, College of Computer and Information Sciences, King Saud University, Riyadh 11543, Saudi Arabia; mmekhtiche@ksu.edu.sa

**Keywords:** surfactant protein D, intermittent hypoxia, obstructive sleep apnea, galectin-3, modified citrus pectin, alveolar integrity, exploratory biomarker

## Abstract

**Highlights:**

**What are the main findings?**

- Intermittent hypoxia induced severity-dependent alterations in circulating surfactant protein D (SP-D), reflecting alveolar epithelial stress.
- Galectin-3 inhibition modulated surfactant homeostasis and reduced cardiopulmonary injury in a context-dependent manner.

**What are the implications of the main findings?**

- SP-D may serve as an exploratory biomarker contributing to severity stratification in obstructive sleep apnea–related hypoxic injury.
- Targeting Galectin-3 signaling represents a potential therapeutic strategy for mitigating intermittent hypoxia–associated lung and cardiac damage.

**Abstract:**

Background: Surfactant Protein D (SP-D) is a critical immunomodulatory collectin maintaining alveolar homeostasis. Obstructive sleep apnea (OSA)-related intermittent hypoxia (IH) disrupts pulmonary surfactant integrity; however, severity-dependent SP-D dynamics remain incompletely characterized. This study explores SP-D as a potential indicator of IH-induced alveolar stress and evaluates whether Galectin-3 (Gal-3) inhibition modulates surfactant homeostasis. Methods: Forty adult male Sprague-Dawley rats (8 per group) were randomized to Control (normoxia), Moderate IH (MIH; 15–30 events/hour), Severe IH (SIH; 30–60 events/hour), MIH + Gal-3 inhibitor (Modified Citrus Pectin, 800 mg/kg/day), or SIH + Gal-3 inhibitor. IH exposure lasted 8 h/day for 10 days. Outcomes included circulating SP-D, Surfactant Protein B (SP-B), inflammatory markers, physiological parameters, and histopathological lung injury scores assessed via American Thoracic Society guidelines. Results: SP-D levels showed numerical reductions with increasing IH severity (Control: 1969.07 pg/mL [IQR: 262.15]; SIH: 1404.30 pg/mL [IQR: 351.88]), representing a 28.6% decrease. However, between-group variability resulted in non-significant omnibus testing (Kruskal–Wallis *p* = 0.187). Gal-3 inhibition elevated SP-D levels, particularly in severe IH (2133.95 pg/mL [IQR: 1240.70]), though high inter-individual variability was observed (CV = 58.1%). SP-B showed significant suppression under moderate IH (*p* = 0.019) with restoration by treatment. Exploratory correlation analysis revealed moderate positive associations between SP-D and heart rate (*r* = 0.587) and respiratory rate (*r* = 0.419) in severe IH, though these did not reach statistical significance (*p* = 0.126 and *p* = 0.301, respectively). Histologically, severe IH induced diffuse alveolar damage (total lung score: 19.67 ± 0.82). Gal-3 inhibition produced context-dependent effects: protective in severe IH but paradoxically exacerbating inflammation under moderate IH (29.20 ± 4.64 vs. 20.00 ± 4.34; *p* < 0.05). Gal-3 inhibition significantly attenuated cardiac injury (injury score: 0.00 ± 0.00 vs. 7.17 ± 0.75 in severe IH; *p* < 0.001, η^2^ = 0.859). Conclusions: SP-D demonstrates severity-associated alterations consistent with alveolar epithelial stress during IH, though high variability limits definitive biomarker validation in this sample. Gal-3 inhibition modulates surfactant homeostasis and attenuates cardiopulmonary injury in a context-dependent manner. These findings support further investigation into SP-D as a component of multimodal severity stratification in OSA and highlight Gal-3 inhibition as a context-dependent anti-inflammatory strategy, pending validation in larger cohorts with tissue-level confirmation.

## 1. Introduction

Obstructive sleep apnea (OSA) impacts nearly one billion adults globally, with epidemiological data demonstrating an increasing prevalence attributed to obesity trends and aging populations [1]. Characterized by repetitive upper airway collapse leading to chronic intermittent hypoxia (IH), this cyclical desaturation–reoxygenation triggers oxidative stress, systemic inflammation, and progressive cardiopulmonary remodeling, establishing OSA as an independent risk factor for heart failure, pulmonary hypertension, and metabolic dysfunction [2,3].

The disruption of alveolar surfactant homeostasis is at the heart of pulmonary problems caused by OSA. Surfactant Protein D (SP-D) is a collagen-containing C-type lectin made mostly by type II pneumocytes and Clara cells. It has two jobs: lowering surface tension and controlling innate immune responses [4]. SP-D attaches to pathogens, dying cells, and inflammatory chemicals, which changes how macrophages work [5]. Clinical studies suggest that circulating SP-D levels may indicate the integrity of the alveolar-capillary membrane; however, the directionality (elevation vs. depletion) is contingent upon acute versus chronic injury patterns [6].

The dynamics of SP-D in chronic IH are still not well understood. Acute hypoxia may enhance surfactant synthesis, whereas chronic hypoxia could modify SP-D via epithelial cell stress, oxidative injury, or depletion during inflammatory conditions [7]. It is not known what the severity-dependent threshold is at which IH affects SP-D, or if this relationship is due to synthetic exhaustion, increased clearance, or changed permeability.

Activated macrophages release galectin-3 (Gal-3), a β-galactoside-binding lectin that is an important upstream regulator of inflammation caused by IH [8]. Increased Gal-3 levels are linked to the severity of OSA and cause pro-fibrotic signaling by activating TGF-β and changing macrophages into the M2 phenotype [9,10]. We posited that Gal-3-mediated inflammation could impair type II pneumocyte function, resulting in variations in SP-D levels, and that pharmacological inhibition of Gal-3 might influence surfactant homeostasis and alveolar architecture.

This study examines SP-D as an exploratory circulating marker potentially reflecting IH-associated alveolar stress, rather than as a validated severity biomarker in a controlled rodent model, analyzing its correlation with physiological cardiopulmonary function and histopathological damage. We additionally assess the therapeutic effectiveness of Gal-3 inhibition (Modified Citrus Pectin) in altering SP-D levels in moderate and severe hypoxic conditions.

## 2. Materials and Methods

### 2.1. Study Design and Setting

This controlled experimental study was conducted between 1 April and 29 July 2025, at the Animal Experimental Center of King Saud University (KSU) in Riyadh, Saudi Arabia. The study protocol simulated OSA by implementing IH, a standard experimental method to replicate the hypoxemia oscillation characteristic of OSA [11]. All procedures followed ARRIVE 2.0 guidelines (Supplementary Table S1) [12].

### 2.2. Experimental Animals and Housing Conditions

Adult male Sprague–Dawley rats (300–400 g body weight, 10–12 weeks old) were obtained from the KSU Animal Experimental Center breeding colony. Animals were specific pathogen-free (SPF) and confirmed healthy by veterinary examination. Rats were housed in standard polycarbonate cages (45 cm × 25 cm × 20 cm; Tecniplast, Buguggiate, Italy) with wood chip bedding (Nestlets^®^, Ancare, Bellmore, NY, USA) under a 12:12 h light:dark cycle at 22–24 °C and 45–65% relative humidity, with ad libitum access to standard laboratory rodent chow (LabDiet 5001, Purina Mills, St. Louis, MO, USA) and filtered tap water. Following a 4-day acclimatization period with daily handling (10 min/day), forty rats were randomized into five experimental groups (*n* = 8 per group; Supplementary Figure S1):

1. Control (CTRLg): Normoxia (ambient air).
2. Moderate IH (MIHg): 15–30 hypoxic events/hour (nadir FiO_2_ 10–12%);
3. Severe IH (SIHg): 30–60 events/hour (nadir FiO_2_ 5–7%);
4. MIH + Gal-3 inhibitor (MIH-Gal-3Ig): Moderate IH + Modified Citrus Pectin (800 mg/kg/day);
5. SIH + Gal-3 inhibitor (SIH-Gal-3Ig): Severe IH + Modified Citrus Pectin (800 mg/kg/day).

Randomization was performed using a computer-generated random number sequence (Microsoft Excel 2019) with allocation concealment via sequentially numbered opaque envelopes. Investigators performing outcome assessments were blinded to group allocation.

### 2.3. Sample Size Calculation

Sample size was determined a priori using G*Power software (v3.1.9.7) [13]. Based on fixed-effects ANOVA with medium effect size (Cohen’s *f* = 0.25), α = 0.05, and power (1 − β) = 0.80, the calculation indicated a minimum of 40 rats (8 per group) to detect between-group differences in cardiopulmonary biomarkers and histopathological endpoints. Biomarker analyses, including SP-D, were considered exploratory outcomes within a study powered primarily for cardiopulmonary injury endpoints.

### 2.4. Intermittent Hypoxia Protocol

IH exposure utilized a custom-built transparent plexiglass chamber (100 cm × 40 cm × 60 cm) with automated gas control (Raspberry Pi 4 system Cambridge, UK), delivering 8 h/day (08:00–16:00) for 10 consecutive days. Hypoxic cycles consisted of 60–120 s at 10% O_2_ (moderate) or 5–7% O_2_ (severe) followed by 30–60 s reoxygenation to 21% O_2_. Chamber oxygen concentrations were monitored continuously using electrochemical oxygen sensors (Gravity I^2^C, Model R-17A, DFRobot/Zhiwei Robotics Corp., Shanghai, China).

### 2.5. Galectin-3 Inhibitor Administration

Galectin-3 (Gal-3) signaling was modulated using modified citrus pectin (MCP), a low-molecular-weight, soluble pectin preparation widely used as an experimental Gal-3 pathway inhibitor because pectin-derived galactose-rich motifs can bind the Gal-3 carbohydrate-recognition domain [14,15]. MCP was used as a commercial product (PectaSol-C^®^, EcoNugenics, Santa Rosa, CA, USA; low molecular weight and low degree of esterification according to manufacturer specification/CoA) and was not produced in-house.

For dosing, MCP powder was prepared fresh daily by dissolving in sterile 0.9% saline with gentle heating to 80 °C (for solubilization only), followed by cooling to room temperature to yield a working concentration of 200 mg/mL. Rats assigned to the inhibitor arms received 800 mg/kg/day MCP by oral gavage (equivalent to ~1.4 mL for a 350 g rat and ~1.5 mL for a 375 g rat at 200 mg/mL), administered 30 min before each daily intermittent hypoxia (IH) session. Treatment began one day prior to IH initiation and continued once daily throughout the 10-day IH protocol (total 11 doses), with dosing performed each morning between 07:00–08:00, including a baseline (Day 0) dose prior to sham handling/exposure.

Vehicle and untreated IH groups received an equivalent gavage volume of sterile 0.9% saline. Protocol compliance and systemic exposure were verified by collecting 0.3 mL tail-vein blood 2 h after the first and last MCP doses and quantifying circulating MCP as uronic acids/galacturonic acid equivalents using a carbazole-based colorimetric assay [15]. Measured concentrations were approximately 50–100 µg/mL, and no adverse reactions attributable to MCP (e.g., aspiration, diarrhea, or acute intolerance) were observed.

### 2.6. Biomarker Quantification

Blood samples were collected at baseline and post-exposure (Day 10) via cardiac puncture under deep anesthesia. Plasma SP-D was quantified using the Rat SP-D ELISA Kit (MyBioSource, San Diego, CA, USA, Cat. No. MBS760898; detection range 0–20 ng/mL). SP-B, IL-6 (Abcam, Cambridge, UK, Cat. No. AB234570), and CRP (Abcam, Cat. No. ab256398) were assessed using commercial ELISA kits per manufacturer protocols.

### 2.7. Physiological Monitoring

Parameters included body weight, systolic/diastolic blood pressure (tail-cuff plethysmography; CODA Monitor, Kent Scientific, Torrington, CT, USA), heart rate (HR), arterial oxygen saturation (SpO_2_), and respiratory rate (RR) (pulse oximetry; Radical-7, Masimo, Irvine, CA, USA).

### 2.8. Histopathological Assessment

At study completion, rats were euthanized under deep anesthesia. Lungs were inflated via tracheal instillation of 10% neutral-buffered formalin at 20 cm H_2_O pressure and fixed for 24–48 h. Hearts were sectioned transversely at the mid-ventricular level and similarly fixed. Tissues were embedded in paraffin and sectioned at 4–5 μm for hematoxylin and eosin (H&E) staining.

Scoring Criteria: Lung injury was evaluated using a semi-quantitative scoring system adapted from American Thoracic Society (ATS) guidelines for experimental lung injury Table 1 [16]. Cardiac injury was assessed using established criteria for myocyte degeneration, interstitial edema, and inflammatory infiltration Table 2 [14].

Sections from five lung lobes (right upper, middle, lower; left upper, lower) and cardiac tissue were evaluated by a veterinary pathologist blinded to group allocation.

### 2.9. Statistical Analysis

Data were expressed as mean ± standard deviation (SD) or median (interquartile range [IQR]) as appropriate. Normality was assessed using Shapiro–Wilk tests. Non-normally distributed variables were analyzed using Kruskal–Wallis tests with Dunn’s post hoc comparisons. Histopathological scores met assumptions of normality and were analyzed using one-way ANOVA with Tukey post hoc testing. Correlations between SP-D and physiological parameters were tested by Spearman methods and designated as exploratory analyses given the sample size (*n* = 8 per group). Statistical significance was set at *p* < 0.05. Analyses were conducted using R Statistical Software (v4.5.1).

## 3. Results

### 3.1. SP-D Levels Show Severity-Associated Trends

**Figure 1:**
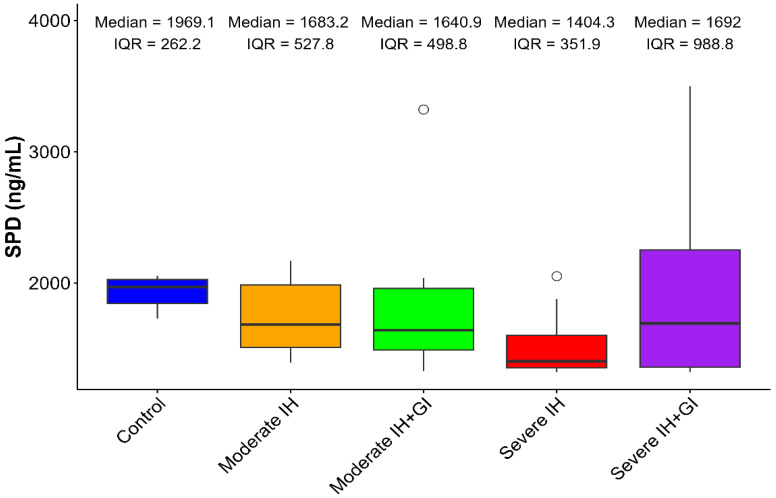
Box plot showing serum Surfactant Protein D (SP-D) levels in the different experimental groups. Data are presented as median and interquartile range (IQR); circles indicate outlier values (Kruskal–Wallis χ^2^ = 6.171, *p* = 0.187).

Circulating SP-D exhibited numerical reductions with increasing IH severity, though between-group variability precluded statistical significance (Kruskal–Wallis χ^2^ = 6.171, *p* = 0.187). Control rats maintained highest levels (median: 1969.07 pg/mL, IQR: 262.15; mean: 1927.6 ± 130.11 pg/mL). Moderate IH showed modest reduction (median: 1683.17 pg/mL, IQR: 527.83; mean: 1739.91 ± 295.87 pg/mL), while severe IH demonstrated the lowest levels (median: 1404.30 pg/mL, IQR: 351.88; mean: 1532.08 ± 278.24 pg/mL), representing a 28.6% numerical decrease from control values (Table 3, Figure 1).

Gal-3 inhibition resulted in elevated SP-D levels, particularly in severe IH (mean: 2133.95 ± 1240.70 pg/mL), though the wide standard deviation indicates substantial variability in treatment response.

### 3.2. Differential Regulation of Surfactant Proteins

In contrast to SP-D trends, SP-B showed significant suppression specifically under moderate IH (median: 1593.84 pg/mL, IQR: 93.77 vs. Control: 1804.00 pg/mL, IQR: 546.36; *p* = 0.019), with restoration by Gal-3 inhibition (1754.68 pg/mL, IQR: 425.17).

### 3.3. Exploratory Correlations with Physiological Parameters

In untreated severe IH, exploratory correlation analysis revealed moderate positive associations between SP-D and both heart rate (*r* = 0.587) and respiratory rate (*r* = 0.419), though these did not reach statistical significance (*p* = 0.126 and *p* = 0.301, respectively). Following Gal-3 inhibition, these associations were abolished (coefficients approaching zero: HR *r* = 0.036; RR *r* = 0.120) Table 4.

### 3.4. Histopathological Validation: Lung Injury Patterns

Severe IH induced diffuse alveolar damage with thickened septa, inflammatory infiltration, and vascular congestion. The right lung and lower lobes showed preferential injury, consistent with gravitational perfusion gradients.

Gal-3 inhibition produced context-dependent biphasic effects. In severe IH, Gal-3 inhibition was associated with reduced regional alveolar injury severity despite similar total lung scores, indicating partial histological protection (total lung score: 22.40 ± 5.06). However, in moderate IH, Gal-3 inhibition was associated with greater histopathological injury under moderate IH condition (29.20 ± 4.64; *p* < 0.05 vs. untreated moderate IH), particularly in right upper (8.20 ± 1.69) and middle lobes (8.60 ± 1.26) Table 5.

### 3.5. Attenuation of Cardiac Injury by Gal-3 Inhibition

**Figure 2:**
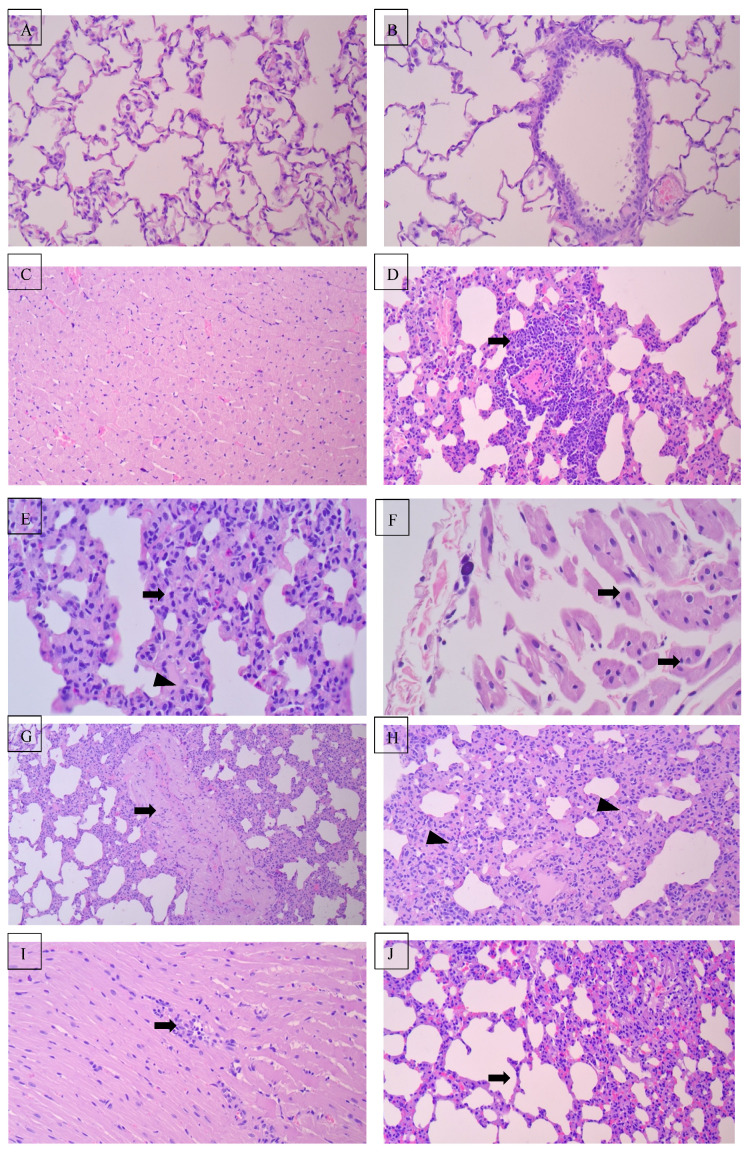

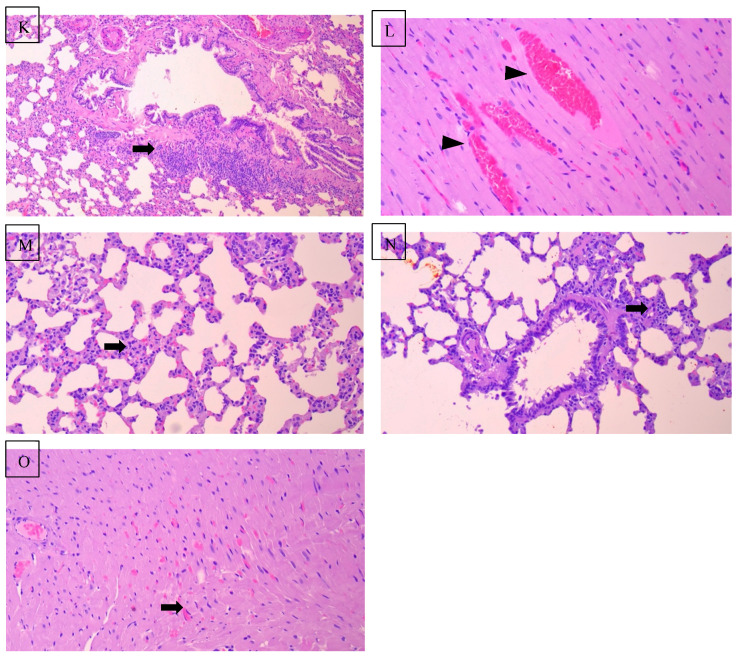
Representative light photomicrographs of H&E-stained lung and cardiac sections (various magnifications). Control group (Cage 3 N2). (**A**) Lung showing well-preserved pulmonary architecture with open alveolar spaces lined by thin pneumocytes and delicate interalveolar septa (×20). (**B**) Bronchiole lined by intact ciliated columnar epithelium resting on a smooth basement membrane, without congestion or inflammation (×20). (**C**) Myocardium displaying regularly arranged cardiac muscle fibers with centrally located oval nuclei, clear cross-striations, and narrow interstitial spaces containing patent capillaries and no evidence of necrosis or inflammation (×20). Moderate interstitial hypertension (G2 B3): (**D**) Bronchiolar wall with mild peribronchiolar mononuclear infiltration and intact ciliated epithelium (arrow) (×40). (**E**) Thickened alveolar septa (arrow) with mild vascular congestion (arrowhead) (×40). (**F**) Myocardial fibers showing capillary congestion and early degenerative changes (arrow) (×40). Severe interstitial hypertension (G3 A3): (**G**) Pulmonary vessel demonstrating marked medial thickening and vascular congestion (arrow) (×10). (**H**) Alveolar septa with dense mononuclear inflammatory infiltration and septal vascular congestion (arrowhead) (×20). (**I**) Myocardium exhibiting interstitial edema, capillary congestion, and degenerating myofibers (arrow) (×20). Moderate IH + GI (G5 A1): (**J**) Lung section showing alveolar septa with reduced thickening and mild vascular congestion (arrow) (×10). (**K**) Bronchiolar wall with mild peribronchiolar inflammatory infiltration and preserved epithelial lining (arrow) (×20). (**L**) Myocardium displaying slight interstitial edema and capillary congestion with preserved fiber morphology (arrowhead) (×20). Severe IH + GI (G4 A3): (**M**) Lung showing mildly thickened alveolar septa with sparse inflammatory cell infiltration and partially open alveolar spaces (arrow) (×20). (**N**) Bronchiole surrounded by mild peribronchiolar inflammatory infiltrate (arrow) with slight epithelial flattening and vascular congestion (×20). (**O**) Myocardium exhibiting mild interstitial edema and focal vascular congestion (arrow) with largely preserved myofibers, reflecting partial attenuation of the severe injury (×200).

Gal-3 inhibition significantly attenuated cardiac injury (injury score: 0.00 ± 0.00 vs. 7.17 ± 0.75 in severe IH; *p* < 0.001, η^2^ = 0.859), with preservation of myocardial architecture and minimal to absent inflammatory infiltrates compared to untreated IH groups. These findings suggest substantial cardioprotective effects of Gal-3 blockade in the setting of chronic IH Figure 2.

## 4. Discussion

### 4.1. Principal Findings

The present study demonstrates three key observations regarding surfactant homeostasis in intermittent hypoxia (IH). First, circulating SP-D exhibited severity-associated numerical reductions (28.6% decrease from control to severe IH), though high inter-individual variability precluded statistical significance (*p* = 0.187). Second, Gal-3 inhibition using Modified Citrus Pectin elevated SP-D levels in severe IH but produced divergent histopathological effects: protective in severe IH yet paradoxically exacerbating alveolar injury in moderate IH. Third, Gal-3 inhibition conferred uniform and marked cardioprotection (complete elimination of histological injury) across both hypoxic paradigms. These findings suggest that while SP-D shows promise as a severity-associated indicator, its utility requires validation in larger cohorts, and Gal-3 inhibition exhibits tissue-specific, context-dependent therapeutic effects.

### 4.2. SP-D Alterations in Chronic IH: Comparison with Clinical Studies

Our observation of reduced circulating SP-D with increasing IH severity aligns with recent clinical investigations identifying SP-D as a sensitive indicator of chronic alveolar stress. Liang et al. [17,18,19] demonstrated that patients with severe OSA exhibit reduced serum SP-D compared to mild-moderate cases, correlating with nocturnal hypoxemia indices. Similarly, Greene et al. [20] reported 30–40% SP-D depletion in idiopathic pulmonary fibrosis, approaching the 28.6% reduction observed in our severe IH group.

However, our findings contrast with acute lung injury models where SP-D elevation reflects epithelial barrier disruption [6]. This divergence supports the hypothesis that chronic IH induces a distinct pathophysiological state characterized by synthetic exhaustion or consumption of SP-D by activated macrophages, rather than acute leakage from damaged pneumocytes. Without bronchoalveolar lavage fluid (BALF) or tissue SP-D quantification, we cannot definitively distinguish between reduced synthesis and increased clearance; however, the restoration of SP-D following Gal-3 inhibition suggests that inflammatory suppression can reverse this depleted state, implicating consumptive processes [21].

### 4.3. Divergent Regulation of Surfactant Proteins

While SP-D showed numerical trends without statistical significance, SP-B exhibited significant suppression under moderate IH (*p* = 0.019) with restoration by Gal-3 inhibition. This differential regulation suggests distinct mechanistic pathways: SP-D appears responsive to chronic inflammatory burden and macrophage activation status, whereas SP-B synthesis appears more acutely sensitive to moderate hypoxic stress and oxidative signaling [22].

This finding contrasts with the coordinated suppression of both proteins observed in acute respiratory distress syndrome [23], suggesting that chronic IH produces a unique surfactant dysregulation pattern. The selective vulnerability of SP-B to moderate hypoxia, combined with its significant response to anti-inflammatory therapy, positions it as a potentially more sensitive early biomarker for moderate OSA, while SP-D may better reflect severe, chronic disease states.

### 4.4. Context-Dependent Pulmonary Effects of Gal-3 Inhibition

Our histopathological findings reveal a paradoxical “Goldilocks” effect of Gal-3 inhibition: while treatment attenuated regional alveolar injury patterns in severe IH, despite comparable total lung injury scores (score 22.40 ± 5.06 vs. 19.67 ± 0.82), it significantly exacerbated damage in moderate IH (29.20 ± 4.64 vs. 20.00 ± 4.34; *p* < 0.05). This aligns with emerging literature suggesting that Gal-3 exhibits biphasic roles in tissue repair. As noted by Bouffette et al. [24] basal Gal-3 signaling facilitates macrophage resolution and remodeling under moderate stress, whereas excessive Gal-3 drives pathological fibrosis under severe stress [25,26].

Our results extend the findings of Mackinnon et al. [27] who demonstrated Gal-3 blockade reduced fibrosis in severe bleomycin injury, by demonstrating that complete inhibition may impair physiological repair mechanisms under moderate stress conditions. This context-dependent response suggests that indiscriminate Gal-3 inhibition in OSA patients without severity stratification could potentially harm those with moderate disease while benefiting severe cases.

### 4.5. Cardioprotective Efficacy and Tissue-Specific Responses

In contrast to the variable pulmonary effects, Gal-3 inhibition produced consistent, histological cardioprotection across both moderate and severe IH (injury score 0.00 ± 0.00 vs. 7.17 ± 0.75; *p* < 0.001, η^2^ = 0.859). This finding supports the cardiovascular literature implicating Gal-3 as a key mediator of hypoxia-induced cardiac remodeling [8,25].

The discrepancy between cardiac and pulmonary responses suggests tissue-specific differences in Gal-3 dependency. Seropian et al. [25] demonstrated, Gal-3 marks activated macrophages in failure-prone hypertrophied hearts, and its inhibition prevents cardiomyocyte dysfunction. Our data indicate that the heart may lack the compensatory repair mechanisms that require basal Gal-3 signaling in the lung, or that the pulmonary system possesses unique inflammatory resolution pathways that are disrupted by complete Gal-3 blockade [25,26].

### 4.6. Physiological Correlations and SP-D as a Stress Indicator

Our exploratory analysis revealed moderate positive associations between SP-D and both heart rate (*r* = 0.587) and respiratory rate (*r* = 0.419) in untreated severe IH, consistent with compensatory cardiopulmonary coupling to maintain oxygenation. This aligns with Lu et al. [26], who demonstrated that SP-D deficiency exaggerates cardiorespiratory responses to hypoxic challenge in mice.

Notably, Gal-3 inhibition abolished these correlations (coefficients approaching zero), suggesting that with inflammatory blockade, SP-D transitions from a stress-responsive indicator to a homeostatic baseline measure. This has diagnostic implications: during anti-inflammatory therapy, SP-D levels may no longer track disease severity, necessitating alternative monitoring strategies.

### 4.7. Clinical Implications and Limitations

Our findings suggest a precision medicine approach wherein circulating SP-D could contribute to severity stratification as part of a multi-biomarker panel, particularly for identifying severe phenotypes characterized by lower SP-D ranges. However, the high variability (CV = 58.1% in treated groups) and borderline statistical significance (*p* = 0.187) require validation in larger cohorts. The paradoxical pulmonary effects of Gal-3 inhibition caution against blanket therapeutic application without severity assessment, while the consistent cardioprotection suggests adjunctive benefit for OSA patients with cardiovascular comorbidities. Additionally, only male rats were studied; therefore, potential sex-specific differences in surfactant regulation and inflammatory responses were not assessed [27].

Several limitations should be considered when interpreting the findings of this exploratory experimental study.

First, circulating SP-D was measured exclusively in plasma, without complementary assessment in lung tissue or bronchoalveolar lavage fluid (BALF). Consequently, the present study cannot definitively distinguish whether the observed reduction in SP-D during severe intermittent hypoxia reflects decreased synthesis by type II pneumocytes, increased pulmonary consumption during inflammatory responses, or enhanced systemic clearance. Surfactant proteins are known to be dynamically regulated at both the tissue and alveolar compartment levels, and plasma measurements alone may not fully reflect pulmonary production or turnover [4,18]. Future studies incorporating tissue-level quantification and BALF analysis will therefore be essential to clarify the mechanistic basis of SP-D alterations during chronic intermittent hypoxia.

Second, although modified citrus pectin (MCP) was administered via oral gavage to ensure accurate dosing and reproducible exposure in the experimental model, pharmacokinetic profiles in rodents may not fully replicate the absorption and metabolism observed with oral MCP supplementation in humans. Experimental studies indicate that MCP exhibits complex bioavailability and systemic distribution characteristics that may vary across species [14,15]. Therefore, the systemic concentrations achieved in this controlled experimental setting may differ from those observed in clinical applications, and the translational implications of MCP therapy should be interpreted with caution until validated in human studies [28].

Third, the study utilized only male Sprague–Dawley rats in order to minimize biological variability associated with sex-dependent hormonal fluctuations, which can influence inflammatory signaling pathways and cardiopulmonary responses to hypoxia. However, obstructive sleep apnea affects both sexes, and emerging evidence suggests potential sex-related differences in inflammatory regulation, surfactant homeostasis, and cardiopulmonary remodeling during hypoxic stress [3,18]. Future investigations incorporating both male and female experimental models are therefore warranted to determine whether sex-specific biological responses influence SP-D dynamics or Galectin-3-mediated inflammatory pathways.

Fourth, although several physiological parameters (including heart rate, respiratory rate, oxygen saturation, and blood pressure) were recorded, the study did not incorporate comprehensive respiratory functional assessments such as pulmonary compliance, airway resistance, or gas-exchange efficiency. As a result, direct correlations between biomarker alterations and objective measures of respiratory functional impairment could not be established. Experimental models of lung injury emphasize that physiological lung mechanics and gas exchange measurements are essential for linking molecular biomarkers with functional respiratory outcomes [16].

Finally, the relatively modest sample size (*n* = 8 per group), while adequately powered for the primary cardiopulmonary injury endpoints, limited statistical power for exploratory biomarker analyses and correlation testing. Statistical interpretation of exploratory biomarker associations in small experimental cohorts should therefore be approached cautiously, particularly when evaluating biological variability and effect size estimation [29]. Larger experimental cohorts will be necessary to validate the observed trends in SP-D variability and to confirm its potential utility as a component of a multimodal biomarker panel for intermittent hypoxia-associated lung injury

### 4.8. Conclusions

This study suggests that SP-D may exhibit exploratory severity-related trends during intermittent hypoxia, supporting its potential inclusion in future multimodal biomarker panels rather than use as a standalone indicator. The findings support continued investigation into surfactant dynamics as indicators of alveolar stress. Gal-3 inhibition exhibits context-dependent pulmonary effects and consistent cardiac protection, underscoring the need for severity-stratified therapeutic approaches in OSA management.

## Figures and Tables

**Table 1:** Histopathological Criteria of Lung Injury (ATS Guidelines).

Score	Histopathological Criteria
0 = Absent	Normal histoarchitecture; no detectable lesion or inflammatory cells
1 = Mild	Focal alveolar wall thickening or scattered inflammatory cells without architectural distortion
2 = Moderate	Multifocal alveolar/interstitial thickening with moderate inflammatory infiltration and vascular congestion
3 = Severe	Diffuse thickening, dense mononuclear infiltration, hemorrhage, or partial alveolar collapse

**Table 2:** Cardiac Injury Scoring Criteria.

Score	Myocardial Alterations
0 = Absent	Normal fibers, no edema or inflammatory cells
1 = Mild	Focal interstitial edema or scattered mononuclear cells
2 = Moderate	Multifocal edema with small clusters of inflammatory cells
3 = Severe	Diffuse edema, vascular congestion, and dense inflammatory infiltration with myocyte degeneration

**Table 3:** SP-D Concentrations Across Study Groups.

Experimental Group	Median (pg/mL)	IQR (pg/mL)	Mean ± SD (pg/mL)	Statistical Significance
CTRLg	1969.07	262.15	1927.6 ± 130.11	χ^2^ = 6.171, *p* = 0.187
MIHg	1683.17	527.83	1739.91 ± 295.87	
SIHg	1404.30	351.88	1532.08 ± 278.24	
MIH-Gal-3Ig	1640.94	498.80	1984.25 ± 973.48	
SIH-Gal-3Ig	1691.97	988.79	2133.95 ± 1240.70	

Note: SIH-Gal-3Ig showed right-skewed distribution (Mean > Median), indicating high inter-individual variability in treatment response (CV = 58.1%).

**Table 4:** Correlation Coefficients **(*r*)** Between SP-D and Physiological Parameters Among Severe IH Groups.

Physiological Parameter	Severe IH (r)	*p*-Value	Severe IH + Gal-3 Inhibitor (r)	*p*-Value
Body Weight	0.108	0.799	0.455	0.257
Systolic Blood Pressure	0.127	0.765	−0.204	0.629
Diastolic Blood Pressure	0.193	0.647	0.192	0.649
Heart Rate	0.587	0.126	0.036	0.933
SpO_2_	0.327	0.429	−0.078	0.854
Respiratory Rate	0.419	0.301	0.120	0.776

Note: Values represent Spearman correlation coefficients. All correlations non-significant (*p* > 0.05). Moderate correlation strength defined as *r* = 0.40–0.60.

**Table 5:** Histopathological Scores (Mean ± SD) Across Experimental Groups.

Tissue/Region	MIHg	SIHg	MIH-Gal-3Ig	SIH-Gal-3Ig	F-Value	*p*-Value
Right Upper Lobe	4.83 ± 2.14 ^a^	4.50 ± 0.84 ^a^	8.20 ± 1.69 ^b^	5.40 ± 1.27 ^a^	10.365	<0.001
Right Middle Lobe	5.50 ± 1.87 ^a^	5.17 ± 0.41 ^a^	8.60 ± 1.26 ^b^	5.40 ± 1.26 ^a^	14.330	<0.001
Right Lower Lobe	4.17 ± 1.83	5.00 ± 0.00	5.00 ± 0.00	5.40 ± 1.27	1.714	0.187
Left Upper Lobe	0.00 ± 0.00	0.00 ± 0.00	0.00 ± 0.00	0.00 ± 0.00	---	---
Left Lower Lobe	5.50 ± 1.76	5.00 ± 0.00	7.40 ± 2.80	6.20 ± 1.93	2.028	0.133
Total Lung Score	20.00 ± 4.34 ^a^	19.67 ± 0.82 ^a^	29.20 ± 4.64 ^b^	22.40 ± 5.06 ^a^	8.925	<0.001
Heart Total Score	4.67 ± 2.94 ^a^	7.17 ± 0.75 ^b^	0.00 ± 0.00 ^c^	0.00 ± 0.00 ^c^	56.870	<0.001

Superscript letters (a, b, c) denote statistically significant differences between groups based on Tukey post hoc comparisons (*p* < 0.05).

## Data Availability

All raw data underlying the findings of this study are available from the corresponding author upon reasonable request.

## Supplementary Materials

The following supporting information can be downloaded at: https://www.mdpi.com/article/10.3390/arm94030027/s1, Figure S1: Experimental design and group allocation of the study; Table S1: ARRIVE 2.0 checklist.
